# Delivery of the 5-HT_2A_ Receptor Agonist, DOI, Enhances Activity of the Sphincter Muscle during the Micturition Reflex in Rats after Spinal Cord Injury

**DOI:** 10.3390/biology10010068

**Published:** 2021-01-19

**Authors:** Jaclyn H. DeFinis, Jeremy Weinberger, Shaoping Hou

**Affiliations:** Marion Murray Spinal Cord Research Center, Department of Neurobiology & Anatomy, Drexel University College of Medicine, Philadelphia, PA 19129, USA; jhd43@drexel.edu (J.H.D.); jw3396@drexel.edu (J.W.)

**Keywords:** micturition, external urethral sphincter, spinal cord injury, serotonin, electromyogram

## Abstract

**Simple Summary:**

Spinal cord injury often disrupts connections between the brain and spinal cord leading to a plethora of health complications, including bladder dysfunction. Spinal cord injured patients are left with symptoms such as a leaky bladder (the inability to hold their urine), frequent urinary tract infections, and potential kidney failure. However, previous studies have shown that manipulation of serotoninergic receptors can improve urinary performance following spinal cord injury. In the current study, we sought to explore how stimulation of a specific serotonergic receptor subtype can significantly enhance bladder function in spinal cord injured rats. To do so, we utilized spinal cord injured female rats that underwent various bladder performance evaluations combined with pharmacological intervention of a specific serotonergic subtype. Additionally, the primary site of action was investigated to determine effects elicited during various administration routes (e.g., directly into the cord, into the femoral vein, or into the skin). Stimulation of this receptor subtype, regardless of delivery route, improved activity of the external urethral sphincter and detrusor-sphincter coordination in spinal cord injured rats. Collectively, the results of these experiments have the potential to provide vital guidance for the development of therapeutic strategies to alleviate urinary dysfunction following spinal cord injury.

**Abstract:**

Traumatic spinal cord injury (SCI) interrupts spinobulbospinal micturition reflex pathways and results in urinary dysfunction. Over time, an involuntary bladder reflex is established due to the reorganization of spinal circuitry. Previous studies show that manipulation of serotonin 2A (5-HT_2A_) receptors affects recovered bladder function, but it remains unclear if this receptor regulates the activity of the external urethral sphincter (EUS) following SCI. To elucidate how central and peripheral serotonergic machinery acts on the lower urinary tract (LUT) system, we employed bladder cystometry and EUS electromyography recordings combined with intravenous or intrathecal pharmacological interventions of 5-HT_2A_ receptors in female SCI rats. Three to four weeks after a T10 spinal transection, systemic and central blockage of 5-HT_2A_ receptors with MDL only slightly influenced the micturition reflex. However, delivery of the 5-HT_2A_ receptor agonist, DOI, increased EUS tonic activity and elicited bursting during voiding. Additionally, subcutaneous administration of DOI verified the enhancement of continence and voiding capability during spontaneous micturition in metabolic cage assays. Although spinal 5HT_2A_ receptors may not be actively involved in the recovered micturition reflex, stimulating this receptor subtype enhances EUS function and the synergistic activity between the detrusor and sphincter to improve the micturition reflex in rats with SCI.

## 1. Introduction

The lower urinary tract (LUT) has two main functions, the storage and periodic elimination of urine. These two processes are dependent upon coordinated activity between the smooth muscle of the bladder detrusor and the striated external urethral sphincter (EUS). In normal conditions, this synergy is accomplished through a complex neural control system involving multiple neurotransmitters and neuropeptides at the brain, spinal cord, and peripheral levels. Among the many neuromodulators of micturition, serotonin (5-hydroxytrpytamine, 5-HT) has gained significant attention over the years as numerous anatomical [1,2,3] and pharmacological investigations [4,5] provide important evidence of 5-HT in the central pathways controlling bladder function [6]. Supraspinal serotonergic pathways mainly originate from the caudal raphe nuclei within the brainstem [7] and distribute terminals to sensory, motor, and autonomic regions of the spinal cord [8]. Additionally, previous studies have demonstrated that 5-HT_2A_ receptors are expressed in the lower spinal cord [9], suggesting that these receptors have a modulatory role in pelvic visceral function.

Traumatic spinal cord injury (SCI) often interrupts spinobulbospinal micturition reflex pathways and results in LUT dysfunction. Following SCI, the bladder is initially areflexic but then partially recovers over time due to the reestablishment of an involuntary spinal micturition reflex pathway [10]. However, this partial recovery entails the emergence of detrusor-sphincter dyssynergia (DSD) and bladder hyperreflexia (or overactivity) which causes inefficient voiding and incontinence. Additionally, a large volume of residual urine remains in the bladder which increases the risk of infection and even life-threatening renal failure [11]. The most common treatments used to empty the bladder or treat overactivity are intermittent catheterization [12], antimuscarinic medications [13], and the neuromuscular blocker onabotulinumtoxin A (BTX-A) [14,15]. However, some treatment strategies have been met with serious health complications. Intermittent catheterization often leads to recurrent urinary tract infections and re-hospitalization, and antimuscarinic medications can have varying effects, such as tachycardia and impairment in cognition depending on what muscarinic receptor they are acting upon [11,16]. Additionally, patients who have undergone BTX-A injections have reported minor side effects, including urinary retention, urinary tract infection, and hematuria [17]. Therefore, limited effective treatment options are available to SCI individuals who suffer from lower urinary tract dysfunction.

The mechanisms that underlie and regulate micturition function after SCI are not well understood. Thus, there is an urgent need to elucidate such mechanisms of the recovered bladder reflex following SCI. Although disrupted supraspinal 5-HT projections no longer transport serotonin to the spinal cord, a small amount of serotonin remains detectable below the lesion [18]. This residual neurotransmitter was believed to be produced solely by sparse intraspinal 5-HT neurons [19]. That is, until recent investigations demonstrated that SCI enables spinal aromatic L-amino acid decarboxylase (AADC) cells distal to the lesion to acquire an enhanced ability to produce 5-HT from its immediate precursor, 5-hydroxytryptophan [20]. This change in spinal AADC cells is thought to be initiated by the loss of inhibition from descending 5-HT projections, which together with an upregulation of 5-HT_2A_ receptors, increases the excitability of spinal motoneurons. In fact, expression of a variety of 5-HT receptors persists in the injured spinal cord [21].

Though previous studies reported that activation of 5-HT_2A_ receptors affects bladder contractions to reduce residual urine and increase voiding efficiency in urethane-anesthetized SCI rats [22], it remains unclear if 5-HT_2A_ receptors regulate activity of the EUS and coordination of detrusor and sphincter activity following SCI. Further, various 5-HT receptor subtypes were recently found to be expressed within the peripheral organs of the LUT [23,24]. It has been suggested that 5-HT_2A_ subtypes can function as postjunctional receptors to peripherally induce detrusor contractions, whereas 5-HT_7_ causes relaxation of the bladder neck [25]. This implies that these receptors may have contrasting roles in micturition function depending on where they are localized. It is thus necessary to differentiate the central vs. peripheral role of the 5-HT system with respect to micturition function. In the present study, we utilized pharmacological interventions of spinal 5-HT_2A_ receptors combined with bladder and EUS reflex assessments as well as metabolic cage assays in conscious SCI rats to answer these questions. The results of these experiments have the potential to provide vital guidance for the development of therapeutic strategies to alleviate urinary dysfunction following SCI.

## 2. Materials and Methods

### 2.1. Animals

A total of 23 female adult Wistar rats (weigh 200–250 g, Charles River, Wilmington, MA, USA) were used. Animals were housed in sets of three per cage to provide social enrichment. Rats with a spinal cord transection (n = 7) were employed for metabolic cages. Some of these rats (n = 6, one suffered a leg injury after testing so it was excluded from subsequent experiments) were later used, together with other SCI rats (n = 6), for micturition reflex assays. Naïve rats (n = 10) were used to collect spinal cord tissue for molecular analyses. Institutional Animal Care and Use Committee and National Institute of Health guidelines on animal care were strictly followed to minimize the number of animals used and any potential suffering. Humane consideration for the well-being of animals was incorporated into the experimental design and conduct. All experimental procedures were reviewed by a local animal care committee to ensure compliance (Project identification code: 1045137).

### 2.2. Spinal Cord Surgery

Rats underwent a complete spinal cord transection at the 10th thoracic level (T10) to remove supraspinal control of micturition. Animals were anesthetized with 2% isoflurane and a partial laminectomy was performed at the T8 vertebrae to expose the dorsal spinal cord. The spinal cord was completely transected at T10 using a No. 11 blade. Lesion completeness was verified visually at the time of surgery. Overlying musculature and skin were then closed. Animals were administered Lactated Ringer’s solution (Baxter Healthcare, Deerfield, IL, USA), cefazolin (10 mg/kg), and buprenex (0.1 mg/kg; Reckitt Benckiser, Slough, United Kingdom) post-operatively. Bladders were manually expressed at least three times per day until sacrifice.

### 2.3. Metabolic Cages

Three weeks after SCI, rats (n = 7) were placed in a metabolic cage apparatus (Braintree Scientific, Chicago, IL, USA) to record spontaneous voiding patterns. It is important to note that prior to being placed in cages, bladders were manually expressed to ensure that they were empty at the beginning of the recording. After being placed into cages, animals were allowed 3 h for acclimation before the administration of drugs or vehicle. Then, animals were subcutaneously (s.c) injected with 300 µL of either saline or the 5-HT_2A_ agonist 2,5-dimethoxy-4-iodoamphetamine (DOI, 60 µg/kg). Urine output was measured on a pressure sensor connected to a computer for the recording of micturition frequency, expelled volume per void of urine, and voiding interval. Data was collected and stored using Windaq-148 software (Dataq Instruments, Akron, OH, USA). This procedure was repeated three times with either saline or DOI injections. The following micturition variables were assessed: (i) total urine expelled, (ii) volume of urine expelled per void, (iii) voiding frequency, and (iv) total water consumed. These variables were evaluated at both a 3 and 6 h time point following drug or vehicle administration. In each rat, parameter values from three different experiments were averaged together to determine the mean value, which was further used to statistically analyze the data using a paired *t*-test.

### 2.4. Bladder Cystometry and EUS Electromyography (EMG) Recordings

Three to four weeks after SCI, rats were anesthetized with 2% isoflurane and an incision was made in the lower abdomen to expose the bladder. The apex of the bladder dome was punctured using a 20-gauge needle. One end of a catheter (PE-60; Clay Adams, New York, NY, USA) was inserted into the bladder [26]. The abdominal wall was then sutured closed with the other end of the catheter protruding from the sutured area to allow for infusion of saline into the bladder. To obtain EUS electromyography (EMG) recordings, the pubic symphysis was removed to expose the urethra and EUS. Two fine-wire electrodes (AstroNova, West Warwick, RI, USA) with exposed tips were percutaneously inserted on both sides of the EUS. Alternatively, two internal electrodes were placed on both sides of the EUS via the opening of the pubic symphysis. In rats receiving intravenous (i.v.) delivery of pharmacological agents (n = 6), a separate cannula (PE-10) filled with saline was implanted in the right femoral vein. In rats receiving intrathecal (i.t.) delivery of drugs or vehicle (n = 6), an ITC 32G catheter (ReCathCo, Allison Park, PA, USA) was placed underneath the dura at the 1st/2nd lumbar spinal cord level (L1/2) and the tip of the catheter was further advanced to the L6/S1 level [27]. In both i.v. and i.t. delivery, rats received injections of gradually increased doses of MDL and then increasing doses of DOI followed by blockage with MDL.

After disconnecting with isoflurane, rats were placed into a restraining cage (KN-326, Natsume, Tokyo, Japan and given time to regain consciousness before recordings. The bladder catheter was connected to a pressure transducer (Transbridge, WPI, Sarasota, FL, USA) and a microinjection pump (SP2001, WPI). Electrodes were connected to an alternating amplifier (P511, AstroNova, West Warwick, RI, USA) and a recording system (Windaq, DATAQ Instruments, Akron, OH, USA) at a sample frequency of 10 kHz. Room temperature saline was slowly infused into the bladder (0.1 mL/min) and at least 1 h adaptation time was ensured before starting the recording. Adaptation was confirmed visually by the appearance of consistent voids as evident in bladder pressure and stabilization of EUS EMG activity. During the recordings, at least 4 continuous stable micturition cycles pre- and post- drug delivery were collected per rat. Experiments lasted no longer than 4 h. Afterwards, the recorded cystometry and EMG traces were opened in Dataq Browser software for analysis. Urodynamic parameters including the voiding amplitude of intravesical pressure (VA), the voiding interval between two sequential voiding events (VI), the voiding volume (VV), bladder contraction duration (CD), and non-voiding contractions (NVCs) were measured. NVCs were defined as rhythmic intravesical pressure increases that were at least 5 mmHg from baseline without a release of fluid from the urethra [28]. Additionally, various EUS EMG parameters were also evaluated including, EUS tonic and bursting activity which occur before and during voiding, respectively. To measure the tonic activity, the root mean square (RMS) and maximum amplitude (MA) of EMG activity were evaluated during 5 s of the filling phase right before a void occurred. The duration of EUS bursting activity during voiding was measured. In these SCI rats, EUS bursting activity occurred ~40–50% of the time while others displayed high-amplitude tonic activity during voiding. In each rat, the parameter values in 4 voiding cycles were averaged to determine the mean value for statistical analysis. The post-drug/saline parameters for each rat were normalized by basal values as a percentage and then a Friedman’s test followed by Dunn’s multiple comparisons was used. Immediately after the recordings, some rats were overdosed with euthasol to harvest fresh tissues for molecular analysis.

### 2.5. Drugs

The 5-HT_2A_ agonist DOI or antagonist (*R*-(+)-α-(2,3-dimethoxyphenyl)-1-[2-(4-fluorophenylethyl)]-4-piperidinemethanol (MDL) were dissolved in saline. The doses of drugs (Table 1 and Table 2) were chosen on the basis of previously described data [22] as well as our own pilot experiments. In metabolic cage assays, DOI (60 µg/kg; 300 µL) was s.c. administered whereas injections of saline (vehicle) served as a control. For cystometry and EUS EMG recordings, saline was delivered i.v. (100 µL) first and increased doses of drugs with equivalent volumes subsequently followed. In rats with i.t. drug administration, a volume of 10 µL was injected for saline or each drug dose. Urodynamic data were collected after each dose of a drug was delivered. The interval was at least 30 min between adjacent doses. For administration of the 2nd drug (DOI), we waited at least 1 h after the last dose of the 1st drug (MDL) was delivered to allow sufficient time for washout. Data were then collected again during basal and saline conditions followed by drug delivery.

### 2.6. Fresh Tissue Harvesting and Protein Isolation

Anesthetized rats were perfused with HEPES buffer and approximately 0.5 cm spinal cord segments at T4/5 above the injury as well as L1/2 and L6/S1 below the injury, where neurons control the LUT, were extracted from naïve (n = 4) and SCI rats (n = 8). Sections were immediately frozen in dry ice and stored in −80 °C. Spinal cords were homogenized in 1 ml of RIPA buffer with Pierce™ protease and phosphatase inhibitors (1 tablet/10 mL RIPA buffer, Thermo Scientific, Waltham, MA, USA). The homogenized spinal cords were centrifuged at 4700 rpm for 90 s, and supernatants were obtained and stored at −80 °C. Following homogenization, standard protein concentrations were established with a Bradford protein assay, and plates were read on an Infinite^®^ 200 Pro microplate reader (Tecan, Männerdorf, Switzerland) at 595 nm using the i-control software package.

### 2.7. Western Blot

A total of 30 μg of each spinal cord sample was denatured using 2× Laemmli buffer containing 5% β-mercaptoethanol at 95 °C for 5 min and loaded onto a Mini-PROTEAN^®^ TGX Stain-Free™ Protein Gel (Bio-Rad, Hercules, CA, USA). All gels ran for 15 min at 80 V, followed by an additional 30 min at 200 V. Following separation, the samples were transferred onto a polyvinylidene difluoride membrane (Bio-Rad) using the Trans-Blot^®^ Turbo™ machine (Bio-Rad). After transfer, membranes were incubated with primary antibody against the 5-HT_2A_ receptor (rabbit, 1:500, Immunostar, Hudson, WI, USA) and glyceraldehye-3-phosphate dehydrogenase (GAPDH, mouse, 1:2000, Cell Signaling Technology, Danvers, MA, USA) overnight at 4 °C. Membranes were then probed with an anti-rabbit or mouse HRP-linked secondary antibody (1:10,000, Cell Signaling). Membranes were then developed with the Clarity™ Western ECL substrate kit (Bio-Rad) and imaged using HyBlot CL™ autoradiography film (Denville, Metuchen, NJ, USA). Receptor expression was normalized to the corresponding protein density of GAPDH. Following normalization, data was averaged and further compared between naïve and SCI groups with a student’s *t*-test (*p* < 0.05).

### 2.8. RNA Extraction/Quantitative Real-Time PCR

Anesthetized rats (n = 6 naïve and 9 SCI) were decapitated and immediately fresh spinal cord tissue at T4/5, L1/2, and L6/S1 segments (approximately 0.5 cm) was dissected. Total RNA was extracted with an E.Z.N.A. Kit II (Omega Bio-Tek, Norcross, GA, USA) according to the manufacturer’s instructions. RNA concentration and the 260/280 nm absorbance ratio were assessed using a Nanodrop spectrophotometer (Thermo Fisher). RNA was reverse transcribed into cDNA using the iScript reverse transcription supermix (Bio-Rad). Quantitative real-time PCR was performed with SYBR Green PCR Master Mix on a CFX Connect Real-Time PCR detection system (Bio-Rad). Primer sequences used were 5-HT_2A_ forward: 5′-AGAAGCCACCTTGTGTGTGA-3′, reverse: 5′-TTGCTCATTGCTGATGGACT-3′; GAPDH forward: 5′-CCATCCCAGACCC CATAAC-3′, reverse: 5′-GCAGCGAACTTTATTGATGG-3′. Expression levels for each amplified gene were calculated using the comparative ΔCt method, where ΔCt = Ct (experimental gene) – Ct (reference gene) for each biological replicate. GAPDH was chosen as a reference gene. For each mRNA measured in qPCR, gene expression values were averaged across biological replicates. The data was further statistically analyzed using a student’s *t*-test to compare gene expression between naïve and SCI rats (*p* < 0.05).

### 2.9. Statistical Analyses

Statistical analyses were performed in GraphPad Prism 8 (GraphPad Software, San Deigo, CA, USA). Significance throughout all experiments was set at *p* < 0.05. Data are represented as mean ± SEM. All groups (intact vs. SCI) and drugs (saline, MDL, DOI) were kept blind to the observer as to avoid biases during molecular and urodynamic analyses. Detailed analyses are listed separately under each experiment described above.

## 3. Results

### 3.1. 5-HT_2A_ Protein and Gene Expression Is Sustained in the Spinal Cord Following SCI

**Figure 1:**
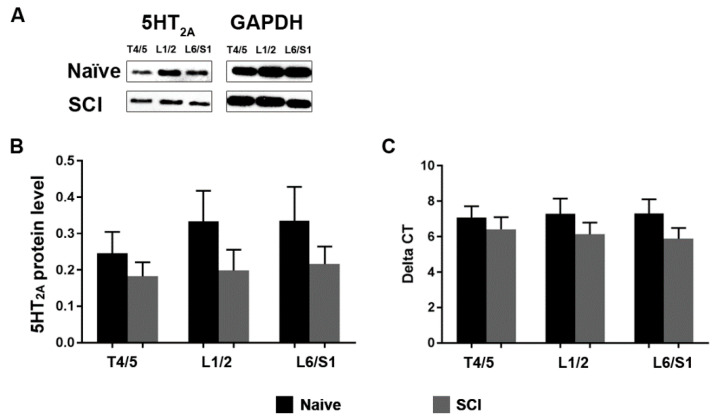
Spinal 5-HT_2A_ receptor expression remains unchanged after SCI. (**A**), Western blots show that there is no difference between naïve and SCI rats in terms of protein expression levels of the 5-HT_2A_ receptor above (T4/5) and below the level of injury (L1/2, L6/S1) (Student’s *t*-test, all *p* > 0.05). (**B**,**C**), Quantitative PCR analysis reveals that mRNA levels of this receptor were not altered at any of the aforementioned segments in SCI rats (all *p* > 0.05). GAPDH served as a control.

Protein expression of 5-HT_2A_ receptors was measured with western blot. No difference was observed between naïve and SCI rats in terms of protein expression levels of this receptor above and below the level of injury (Naïve T4/5, 0.24 ± 0.05; SCI T4/5, 0.18 ± 0.03; Naïve L1/2, 0.33 ± 0.08; SCI L1/2, 0.19 ± 0.05; Naïve L6/S1, 0.33 ± 0.09; SCI L6/S1, 0.21 ± 0.04; all *p* > 0.05, Unpaired *t*-test). This included two important segments, L1/2 and L6/S1, which are related to micturition control (Figure 1A, B). In line with the aforementioned results, mRNA levels of 5-HT_2A_ receptors measured with qPCR were not altered (Naïve T4/5, 7.08 ± 0.62; SCI T4/5, 6.41 ± 0.68; Naïve L1/2, 7.28 ± 0.85; SCI L1/2, 6.13 ± 0.65; Naïve L6/S1, 7.30 ± 0.79; SCI L6/S1, 5.89 ± 0.59; all *p* > 0.05) in SCI rats (Figure 1C). Therefore, the results suggest that expression of 5-HT_2A_ receptors is sustained in the spinal cord after SCI.

### 3.2. I.v. Drug Delivery and its Effects on Bladder and EUS Reflexes

**Figure 2:**
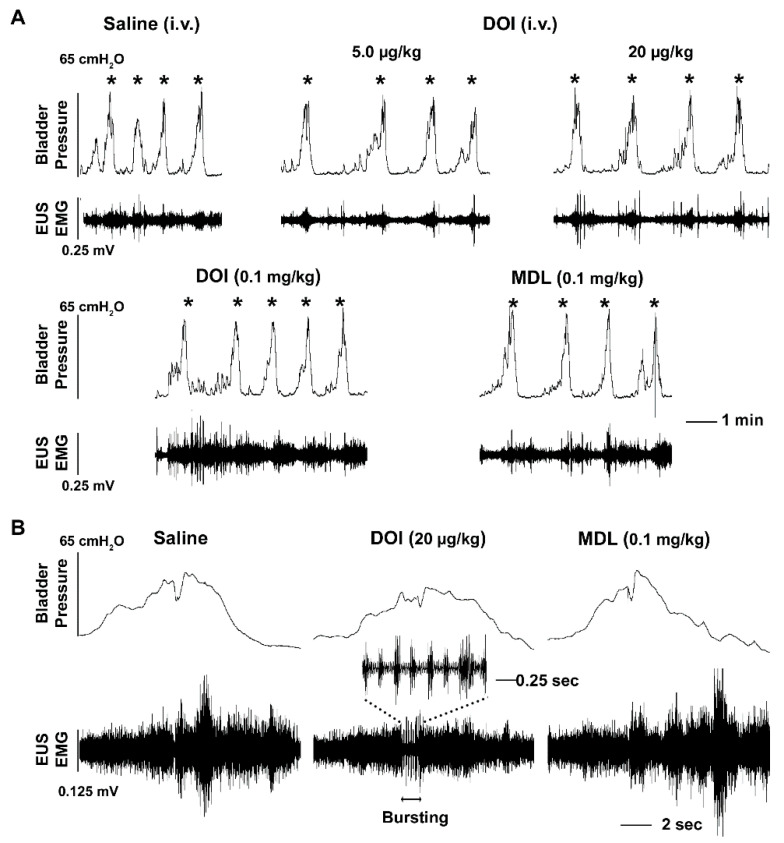
Intravenous (i.v.) administration of DOI to stimulate 5-HT_2A_ receptors improves the micturition reflex during bladder cystometry and sphincter EMG in SCI rats. (**A**), Stimulating 5-HT_2A_ receptors with the middle dose of DOI (20 µg/kg) prolongs the VI (*p* < 0.05, Friedman’s test followed by Dunn’s) between voiding contractions (asterisks). The high dose of DOI (0.1 mg/kg) increases EUS tonic activity in the filling phase (*p* < 0.01). Ensuing injection of MDL (0.1 mg/kg) eliminates the provoked effects. (**B**), Representative traces show no EUS bursting activity during voiding when saline is delivered. However, the middle dose of DOI triggers EUS bursting activity during voiding. Injection of MDL following the high dose of DOI masks the triggered bursting EUS activity during voiding.

Three to four weeks after T10 spinal cord transection, animals were utilized for bladder cystometry and EUS EMG assays. Firstly, a serial dose of the 5-HT_2A_ receptor antagonist MDL was administered i.v. to determine the function of these receptors in the recovered micturition reflex (Table 1). Following administration of the antagonist, there were no dramatic changes in cystometry and EMG parameters except a significantly prolonged VI (*p* < 0.05, Friedman’s test followed by Dunn’s) following injection of the middle dose of the drug. However, some obvious responses were detected after the 5-HT_2A_ receptor agonist DOI was administered. The mid (20 µg/kg) dose of the drug was able to increase the VI (*p* < 0.05) and induce a larger VV (*p* < 0.01). The occurrence of NVCs had no significant changes after administration of any of the drugs (all *p* > 0.05). Notably, the high dose of DOI (0.1 mg/kg) produced a significant increase in tonic EUS activity (RMS *p* < 0.01) and there was also a trend for an increase in the MA value. It also induced non-specific activity of the EUS along with high amplitude contraction during continuous infusion of saline into the bladder (Figure 2A). More importantly, DOI was able to trigger a bursting pattern in the EUS in 4 rats that did not have any such events during baseline or saline delivery. It is important to note that corresponding bladder high frequency oscillations (HFO) did not often accompany bursting in these animals (Figure 2B). Ensuing administration with the mid dose of MDL (0.1 mg/kg) abolished the effects of DOI on the EUS, including decreased EUS tonic activity in the filling phase and masked bursting during voiding. Specifically, the results illustrate that systemic blockage of 5-HT_2A_ receptors with MDL has subtle effects while activation of this receptor subtype with DOI increases EUS tonic activity and elicits bursting to facilitate voiding in SCI rats.

### 3.3. I.t. Drug Delivery and Its Effects on Bladder and EUS Reflexes

**Figure 3:**
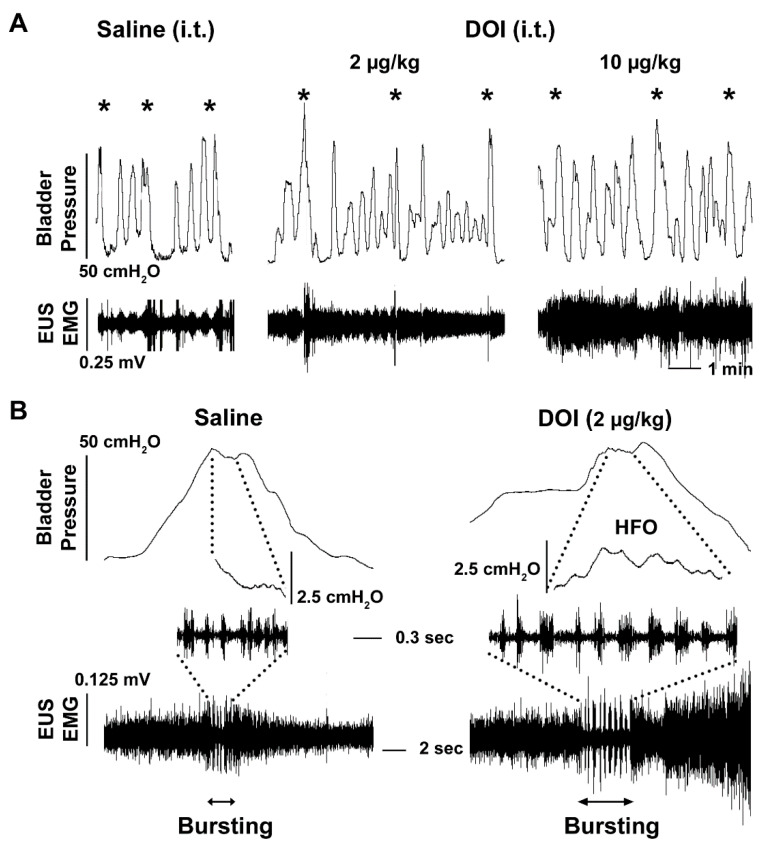
Intrathecal (i.t.) administration of DOI to stimulate 5-HT_2A_ receptors affects the micturition reflex during bladder cystometry and sphincter EMG in SCI rats. (**A**), Representative tracers show that stimulating 5-HT_2A_ receptors with the middle (2 µg/kg) and high (10 µg/kg) doses of DOI prolongs the VI (both *p* < 0.05, Friedman’s test followed by Dunn’s) between voiding contractions (asterisks) and induces an increase in EUS tonic activity. (**B**), Importantly, the middle dose of DOI increases the duration of EUS bursting that consists of more regularly occurring active and silent periods along with bladder HFO, indicating an enhancement of detrusor-sphincter coordination during voiding.

**Figure 4:**
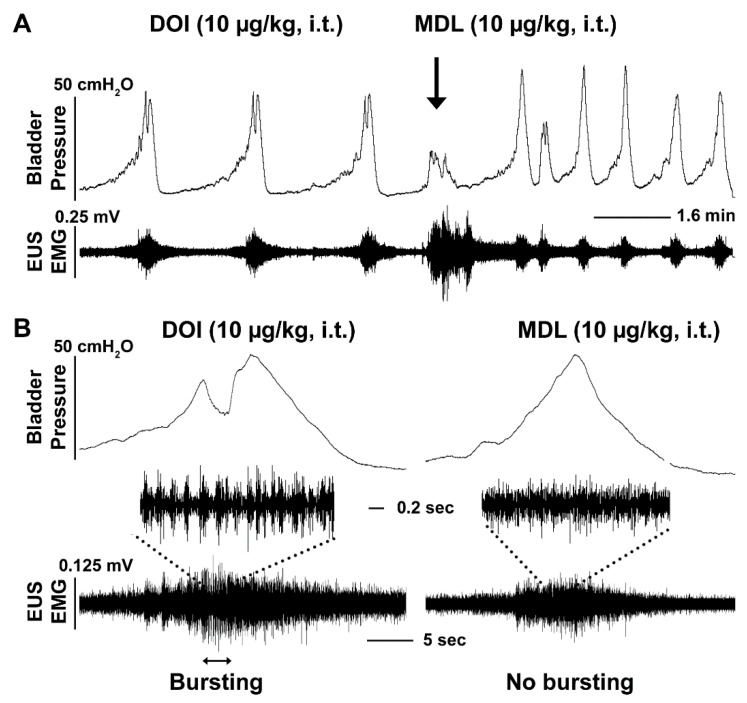
Intrathecal (i.t.) administration of MDL abolishes DOI induced excitatory effects on the EUS reflex in SCI rats. A representative cystometry and EMG trace demonstrates that MDL, a 5-HT_2A_ receptor antagonist (10 µg/kg), shortens the VI (*p* < 0.05, Friedman’s test followed by Dunn’s) (**A**) and masks bursting EUS activity (**B**), leading to the occurrence of detrusor-sphincter dyssynergia.

To clarify whether the effects produced from 5-HT_2A_ receptor manipulation on the urinary system were working through central mechanisms, we administered drugs i.t. during bladder cystometry and EUS EMG assays in SCI rats (Table 2). As a result, central delivery of the 5-HT_2A_ antagonist MDL did not trigger significant responses in bladder and EUS reflexes. When DOI was administered, both the middle and high doses significantly prolonged the VI (both *p* < 0.05, Friedman’s test followed by Dunn’s). The high dose increased the VV (*p* < 0.05). Particularly, there was a trend for the high dose of DOI to induce an increase in EUS tonic activity in both the RMS (1.4 fold) and MA (1.6 fold) values, although not statistically significant. During voiding, EUS bursting activity accompanied by bladder detrusor HFO were elicited in 4 animals that did not have such events originally. The middle dose of DOI increased the duration of EUS bursting (saline 1.6 ± 0.9, DOI 3.8 ± 1.5 s) in 2 rats that had such activity during baseline recordings. This suggests that DOI can improve the synergistic activity of the sphincter and detrusor (Figure 3). In addition, there was a trend that suggests the number of NVCs increases after the middle and high doses of DOI injections, which could be related to the dramatically prolonged VI seen with these two doses. After administration with the middle dose of MDL, the effects of DOI on the EUS were completely abolished. This was apparent by the presence of reduced EUS tonic activity and the disappearance of bursting (Figure 4). MDL also shortened the VI of bladder voiding contractions. This suggests that activation of 5-HT_2A_ receptors, by central administration of DOI, largely affects activity of the EUS, leading to improved micturition function in SCI rats.

### 3.4. S.c. Delivery of DOI Improves Spontaneous Micturition Performance Following SCI

**Figure 5:**
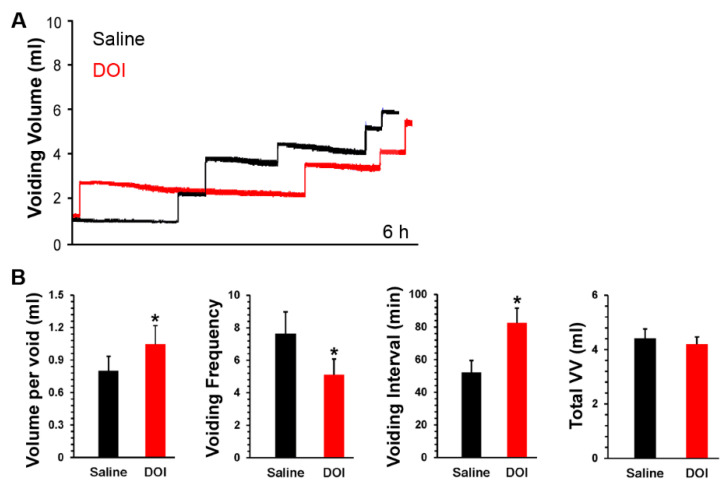
Stimulating 5-HT_2A_ receptors with DOI delivery improves spontaneous micturition function in SCI rats. Representative traces show volume-frequency patterns of urination in metabolic cage assays after 5-HT_2A_ receptor stimulation with DOI within a 6-h time period. Saline injections served as a control. In each “step-like” curve of these traces (**A**), the vertical lines represent the VV per void and the horizontal lines illustrate the VI. After s.c. injection of the 5-HT_2A_ receptor agonist DOI (60 µg/kg, 300 µL), (**B**) there was a significant increase in the VV per void and VI in comparison to saline controls (Paired *t*-test, all * *p* < 0.05).

SCI rats were s.c. administered DOI in metabolic cage assays. During the 6 h time period following drug delivery, DOI significantly increased the VI (saline 52.19 ± 7.36, DOI 82.60 ± 8.93; Paired *t*-test, *p* < 0.05) and VV per void (saline 0.8 ± 0.13, DOI 1.04 ± 0.17, *p* < 0.05) but decreased the voiding frequency (saline 7.63 ± 1.33, DOI 5.11 ± 0.95, *p* < 0.05) (Figure 5). It is important to note that urodynamic parameters were examined out to 12 hr post-drug delivery. However, it was found that only significant changes in urinary performance could be detected at a maximum of 6 h post-drug administration. There was no significant change in the total VV. Total water intake was not significantly different between drug and saline controls (both *p* > 0.05). This implies that DOI can improve spontaneous micturition function by increasing continence and voiding capability.

## 4. Discussion

The present study examined the contribution of 5-HT_2A_ receptors in the micturition reflex after SCI, with a particular focus on the function of these receptors in the EUS. Blocking spinal 5-HT_2A_ receptors with central delivery of MDL did not have a meaningful impact on bladder and sphincter activity. This denotes that endogenous spinal 5-HT, which has been shown to be present at low levels in the cord after injury, [18] may not modulate recovered micturition function via spinal receptors following SCI. However, systemic or central stimulation of 5-HT_2A_ receptors with DOI induced similar effects that increased EUS tonic activity in the filling phase and triggered bursting during voiding. This indicates that DOI enhances synergistic activity between the detrusor and sphincter to improve voiding capability. Thus, DOI elicits its effects mainly through spinal receptors regardless of delivery route. Though spinal 5-HT_2A_ receptors may not play a role in the established involuntary spinal micturition reflex after SCI, our results suggest that stimulation of these receptors mainly acts on motoneurons controlling the EUS to improve coordination of bladder and sphincter activity while also mitigating DSD to enhance micturition function.

Serotonergic regulation of LUT function has been explored since the 1960s when dense supraspinal 5-HT innervation to autonomic nuclei was observed in the lower spinal cord [29,30], including the dorsal horn and Onuf’s nucleus which contains the motoneurons that control EUS function [7,31]. Although SCI interrupts descending neuronal pathways, previous studies have demonstrated the ability of the injured spinal cord to produce monoamines, such as serotonin, caudal to the level of injury [20]. Further, the expression of various serotonergic receptors within the lumbosacral cord has been examined [32,33] as well as an indication for their role in micturition function [34,35,36,37,38]. Accordingly, it is possible that these spinally located 5-HT receptors also serve to modulate pelvic visceral function after SCI. In line with this, our results have confirmed that neither the protein nor mRNA levels of 5-HT_2A_ receptors significantly decreased above or below the level of injury. However, previous studies reported that their expression is upregulated in motoneurons following SCI [39]. This contradiction could be due to the fact that our tissue samples were collected from spinal segments as opposed to specific populations of individual neurons. Nevertheless, spinal 5-HT_2A_ receptor expression is sustained in the cord of SCI rats.

Serotonergic receptors lie within both the central and peripheral nervous systems. Despite the fact that 5-HT_2A_ receptors are widely distributed throughout the brain and spinal cord [9,31,40], this subtype is also present in the smooth muscle and urothelium of the bladder [24]. This implies that systemic drug administration may have both peripheral and central-mediated effects. The middle dose of MDL increased the VI when it was i.v. injected but did not do so in i.t. delivery, suggesting this minor effect may be mediated peripherally. In opposition to this, delivery of DOI both i.v. or i.t. elicited similar effects on the micturition reflex. The role of DOI in bladder function following SCI has previously been studied and dramatic effects on the facilitation of voiding were reported. Yet, it is unknown if this drug acts on motoneurons controlling the EUS muscle with regards to the coordination of the detrusor and sphincter for micturition function.

In the present study, DOI was able to elicit EUS bursting and bladder HFO. This indicates that stimulating spinal 5-HT_2A_ receptors, irrespective of delivery route, largely triggers its effects through central mechanisms that control the sphincter muscle. The EUS is continuously relaxed during voiding in humans whereas it is phasically relaxed in rats. Bursting EUS activity and bladder HFO occur during the expulsion phase in rats. SCI rats mainly show increased EUS tonic activity with little or no bursting and virtually no bladder HFO during voiding [41]. EUS bursting consists of both high frequency spikes that are deemed active periods (AP) and quiescent periods known as silent periods (SP), which alternate in such a way to allow for relaxation and contraction of the EUS, respectively, and are essential for efficient voiding [42]. HFO are thought to be generated by EUS bursting activity since these two patterns often occur synergistically [43]. Here we reported that stimulation of spinal 5-HT_2A_ receptors with DOI provoked EUS bursting. Subsequently, delivery of MDL to block these receptors eliminated the aforementioned effects, confirming mediation by spinal 5-HT_2A_ receptors. Similar to results from previous studies in which 5-HT_2A_ receptors exert excitatory control of the EUS in spinal cord intact rats [44], our results verified that activation of spinal 5-HT_2A_ receptors in SCI rats triggers EUS bursting which originates from simultaneous firing of Onuf’s motoneurons. Nevertheless, it has been assumed that propriospinal neurons in the L3/4 spinal cord contribute to the emergence of EUS bursting and bladder-EUS coordination [45]. If this is the case, DOI may indirectly affect Onuf’s motoneurons or does so via propriospinal neurons that express 5-HT_2A_ receptors.

Stimulation of spinal 5-HT_2A_ receptors with DOI increased EUS tonic activity in the filling phase during reflex assessments. Since EUS tonic activity reflects the capability of continence and DOI prolonged this activity between bladder contractions (e.g., prolonged VI), our data implies that DOI augments the ability to prevent leakage. This was also confirmed in spontaneous micturition assays by metabolic cages and s.c. administration of DOI in SCI rats. Because DOI did not affect the amplitude of bladder contractions, it is reasonable to conclude that the general impact of DOI on the micturition reflex is primarily caused by the agonist’s effects on motoneurons controlling the EUS rather than on parasympathetic preganglionic neurons regulating the bladder. In addition to its effects on the EUS, stimulation of 5-HT_2A_ receptors with DOI was able to significantly increase the voiding volume with both i.v. and i.t. delivery. This implies that the drug improved voiding efficiency by either (1) reducing the residual volume of urine that remains in the bladder following a contraction, (2) increasing bladder capacity, or (3) both. Based on previous reports, delivery of DOI peripherally or centrally increases the voiding volume while reducing the residual volume of urine [22,39]. Therefore, it seems likely that the increase in the voiding volume illustrated in the current experiments is due to improved voiding efficiency. It appears that DOI can produce similar effects in LUT performance when administered intravenously, intrathecally, and even subcutaneously. Thus, administration of DOI mainly stimulates centrally located 5-HT_2A_ receptors and contributes to detrusor-sphincter synergy to enhance voiding capability in SCI rats. Additionally, it is known that DOI also has an affinity for 5-HT_2C_ receptors [46,47]. Previous studies have shown that central 5-HT_2C_ receptors mainly inhibit the micturition reflex [4]. With this in mind, we administered MDL, which is a selective antagonist for 5-HT_2A_ receptors [48], directly following the high dose of DOI. This ultimately blocked changes that DOI induced on urinary function (e.g., EUS bursting and bladder HFO), and therefore, further supports the notion that DOI improves micturition performance via 5-HT_2A_ and not _2C_ receptors.

Urethane is currently the most widely used anesthetic to study micturition physiology. In fact, the majority of studies that examine the role of 5-HT receptors have been completed with the use of urethane or some other anesthetic [22,37,49]. The widespread use of this anesthetic is largely due to the fact that it spares the micturition reflex and is long-lasting [50]. Although urethane preserves vital parameters, such as bladder HFO and EUS bursting, it can interfere with the actions of glutamate and its analogs in the micturition pathway [51]. In addition, urethane was found to suppress sympathetic outflow to the bladder, and thus alter bladder capacity [52]. Notably, things become more complicated when applying anesthetics to SCI animals that already display several urinary complications (e.g., no bursting periods and emergence of non-voiding contractions) [53]. Therefore, the present study sought to avoid analgesic-related confounds by using awake rats for bladder cystometry and EUS EMG recordings.

Although most SCI patients have incomplete injuries as opposed to complete [54,55], a complete spinal cord transection model was deemed the most appropriate for the purposes of this study. The overall goal of this research was to determine the role of 5-HT_2A_ receptors in the recovered involuntary micturition reflex following SCI, specifically as it pertains to EUS activity since this has not been thoroughly examined. If an incomplete injury was to be used in the current experiments, it would be almost impossible to differentiate whether the agonist or antagonist were acting at both supraspinal and spinal levels that control bladder and EUS reflexes due to sparing. Accordingly, to focus on spinal serotonergic mechanisms, it was necessary to disrupt all supraspinal control. However, future experiments may be conducted in a contusion model to determine the efficacy of the effects seen within the current experiments with DOI. Additionally, we used female rats instead of males due to the ease of bladder care. Since male rats have a long and narrow urethra [56], bladder care is very difficult in these animals directly following injury. Importantly, our previous work has shown that both males and females manifest similar urinary complications, such as DSD and bladder hyperreflexia, following SCI. However, future experiments should be conducted in male SCI rats to confirm that there are no apparent sex differences in terms of the role that 5-HT_2A_ receptors play in the recovered bladder reflex.

In clinical studies, it was established that duloxetine, a 5-HT and norepinephrine reuptake inhibitor, was able to significantly reduce stress-related urinary incontinence episodes in women [57]. Duloxetine is proposed to improve continence by increasing the bioavailability of 5-HT and norepinephrine at presynaptic neurons in Onuf’s nucleus of the sacral cord, which in turn, is thought to increase EUS activity. This suggests that serotonergic mechanisms also exist in the human spinal cord, and stimulating these pathways enhances LUT performance not only following SCI but across a broad range of conditions that elicit urinary complications. Nevertheless, it is important to note that the effects of each drug used within our study do not discern what population of cells these receptors are acting upon. Thus, sites of action should be further explored by whole cell patch clamp recordings in future studies.

## 5. Conclusions

Endogenous spinal 5-HT may not modulate recovered micturition function via 5-HT_2A_ receptors following SCI. Nonetheless, both systemic and central stimulation of these receptors with DOI can induce similar effects to increase EUS tonic activity in the filling phase and trigger bursting during voiding. It indicates that DOI enhances coordination of detrusor and sphincter activity to improve micturition function in SCI rats.

## Figures and Tables

**Table 1:** Normalized parameters of bladder cystometry and EUS EMG activity in SCI rats (*i.v.* administration).

Drugs	Doses (/kg)	Bladder CMG				EUS EMG		VV
		VA	CD	VI	^#^ NVCs	RMS	MA	
MDL (n = 6) (*5HT_2A_ receptor antagonist*)	*Saline*	1.11 ± 0.10	0.96 ± 0.05	1.00 ± 0.05	1.31 ± 0.31	0.95 ± 0.06	0.82 ± 0.12	0.95 ± 0.09
	20 µg	1.01 ± 0.19	0.93 ± 0.05	1.07 ± 0.14	1.25 ± 0.70	0.87 ± 0.02	0.71 ± 0.09	1.00 ± 0.15
	0.1 mg	0.97 ± 0.06	0.98 ± 0.06	1.22 ± 0.11 *	1.56 ± 0.86	0.96 ± 0.09	0.81 ± 0.10	1.15 ± 0.10
	0.5 mg	1.04 ± 0.04	0.95 ± 0.06	1.10 ± 0.07	0.88 ± 0.79	1.11 ± 0.16	1.00 ± 0.37	1.09 ± 0.14
DOI (n = 6) (*5HT_2A_ receptor agonist*)	*Saline*	1.11 ± 0.10	0.96 ± 0.05	1.00 ± 0.05	1.31 ± 0.31	0.95 ± 0.06	0.82 ± 0.12	0.95 ± 0.09
	5.0 µg	1.08 ± 0.04	0.98 ± 0.04	1.34 ± 0.11	1.00 ± 0.84	1.03 ± 0.14	0.69 ± 0.97	1.23 ± 0.15
	20 µg	1.06 ± 0.08	1.12 ± 0.04	1.73 ± 0.17 *	2.19 ± 1.59	1.13 ± 0.13	0.84 ± 0.21	1.32 ± 0.12 *
	0.1 mg	1.00 ± 0.08	1.01 ± 0.04	1.51 ± 0.30	1.31 ± 0.80	1.53 ± 0.24 *	1.64 ± 0.55	1.24 ± 0.17

* *p* < 0.05; VA, voiding amplitude of intravesical pressure, CD, contraction duration, VI, voiding interval, VV, voiding volume, NVCs, non-voiding contractions, RMS, root mean square, MA, maximum amplitude of tonic activity; ^#^ non-normalized data.

**Table 2:** Normalized parameters of bladder cystometry and EUS EMG activity in SCI rats (*i.t.* administration).

Drugs	Doses (/kg)	Bladder CMG				EUS EMG		VV
		VA	CD	VI	^#^ NVCs	RMS	MA	
MDL (n = 6) (*5HT_2A_ receptor antagonist*)	*Saline*	1.23 ± 0.11	1.01 ± 0.01	1.12 ± 0.11	0.29 ± 0.15	0.97 ± 0.19	0.92 ± 0.07	0.81 ± 0.03
	2 µg	1.13 ± 0.10	0.97 ± 0.01	1.12 ± 0.11	0.20 ± 0.13	1.09 ± 0.06	0.95 ± 0.08	0.78 ± 0.03
	10 µg	1.22 ± 0.18	1.08 ± 0.09	1.34 ± 0.20	0.29 ± 0.15	0.98 ± 0.05	0.74 ± 0.11	1.07 ± 0.09
	50 µg	0.81 ± 0.08	0.83 ± 0.05	1.19 ± 0.15	0.58 ± 0.26	1.11 ± 0.05	1.46 ± 0.30	0.95 ± 0.09
DOI (n = 6) (*5HT_2A_ receptor agonist*)	*Saline*	0.94 ± 0.03	1.09 ± 0.04	1.24 ± 0.14	1.08 ± 0.60	1.01 ± 0.03	1.17 ± 0.20	0.95 ± 0.10
	0.5 µg	1.03 ± 0.09	1.14 ± 0.05	1.42 ± 0.46	0.79 ± 0.64	1.01 ± 0.03	1.05 ± 0.09	0.88 ± 0.12
	2 µg	1.21 ± 0.13	1.13 ± 0.08	3.27 ± 1.04 *	2.70 ± 1.77	1.14 ± 0.17	1.12 ± 0.28	1.14 ± 0.18
	10 µg	1.12 ± 0.11	1.09 ± 0.05	3.98 ± 1.58 *	3.33 ± 2.41	1.46 ± 0.21	1.96 ± 0.31	1.33 ± 0.18 *

* *p* < 0.05; VA, voiding amplitude of intravesical pressure, CD, contraction duration, VI, voiding interval, VV, voiding volume, NVCs, non-voiding contractions, RMS, root mean square, MA, maximum amplitude of tonic activity; ^#^ non-normalized data.

## Data Availability

The data that support the findings of this study are available from the corresponding author, (Shaoping Hou, PhD), upon reasonable request.
